# Evapotranspiration estimation using Surface Energy Balance Model and medium resolution satellite data: An operational approach for continuous monitoring

**DOI:** 10.1038/s41598-023-38563-2

**Published:** 2023-07-25

**Authors:** S. Pareeth, P. Karimi

**Affiliations:** Department of Land and Water Management, IHE Delft Institute for Water Education, 2611 AX Delft, The Netherlands

**Keywords:** Environmental impact, Hydrology

## Abstract

Monitoring spatial and temporal trends of water use is of utmost importance to ensure water and food security in river basins that are challenged by water scarcity and climate change induced abnormal weather patterns. To quantify water consumption by the agriculture sector, continuous monitoring is required over different spatial scales ranging from field (< 1 ha) to basin. The demand driven requirement of covering large areas yet providing spatially distributed information makes the use of in-situ measurement devices unfeasible. Earth observation satellites and remote sensing techniques offer an effective alternative in estimating the consumptive use of water (Actual EvapoTranspiration (ET_a_) fluxes) by using periodic observations from the visible and infrared spectral region. Optical satellite data, however, is often hindered by noises due to cloud cover, cloud shadow, aerosols and other satellite related issues such as Scan Line Corrector (SLC) failure in Landsat 7 breaking the continuity of temporal observations. These gaps have to be statistically filled in order to compute aggregated seasonal and annual estimates of ET_a_. In this paper, we introduce an approach to develop a gap-filled multi-year monthly ET_a_ maps at medium spatial resolution of 30 m. The method includes two major steps: (i) estimation of ET_a_ using the python based implementation of surface energy balance model called PySEBAL and (ii) temporal interpolation using Locally Weighted Regression (LWR) model followed by spline based spatial interpolation to fill the gaps over time and space. The approach is applied to a large endorheic Lake Urmia Basin (LUB) basin with a surface area of ~ 52,970 km^2^ in Iran for the years 2013–2015 using Landsat 7 and 8 satellite data. The results show that the implemented gap filling approach could reconstruct the monthly ET_a_ dynamics over different agriculture land use types, while retaining the high spatial variability. A comparison with a similar dataset from FAO WaPOR reported a very high correlation with R^2^ of 0.93. The study demonstrates the applicability of this approach to a larger basin which is extendible and reproducible to other geographical areas.

## Introduction

Water scarcity is one of the most critical global issues of the twenty-first century which has affected the daily livelihood of millions. An estimated forty percent of the global population are experiencing water scarcity at different magnitudes^1,2^. In Asia and the Middle East region, water scarcity is mainly driven by climate change, improper water use, and growing population^3^. Many studies have reported a rapid decrease in the level of groundwater table in the semi-arid regions of Asia and the Middle East mainly due to the overuse of water to meet irrigation needs^4–6^. Therefore, there is a great need to establish monitoring systems to facilitate timely interventions ensuring sustainable management of land and water resources. One of the Sustainable Development Goal (SDG)—SDG 6 targets to substantially reduce the number of people suffering from water scarcity by improving water use efficiency by the year 2030^7^. The biggest share of water (around 70%) in Asia is allocated for irrigation^8^. Hence, to improve water use efficiency it is crucial to monitor the water use from the basin scale where water allocation to different sectors takes place^9,10^ to the field scale where the water saving interventions are often deployed^11^. The requirement of covering large geographical areas, yet providing very fine resolution information makes the use of in-situ measurement devices unfeasible due to technological limitations as well as the large commitment required in terms of equipment costs and maintenance.

Remotely sensed information from satellites plays a crucial role in hydrology and water resources management, allowing for monitoring and assessment of surface water bodies, land and water use, precipitation patterns, and soil moisture levels over large spatial scales. It is considered as a viable alternative to in-situ data for monitoring land and water use in agriculture^12^. Frequent observations of satellite data in visible and infrared spectral region has been developed to be a cost-effective, scalable, and reliable data source for measuring ET_a_^13^. In recent decades, the utilization of remote sensing has significantly expanded in terms of spectral and temporal resolution. This growth can be attributed to the launch of new satellite/sensor missions, the adoption of open data policies by agencies, and the advancement of algorithms used to accurately extract land use and geophysical parameters^14^. In agriculture, there is an increasing demand to use remotely sensed information to monitor and assess water use and irrigation performances at different scales from basin to field level^9^. Remote sensing can be used as a valuable source of information for water resource planners to conduct water accounting^15^ and for irrigation managers to estimate the spatial distribution of water use and productivity^16^. Satellite data offers unique spatial and temporal coverage with sensors like Moderate Resolution Imaging Spectroradiometer (MODIS) (with ~ 250–1000 m spatial and daily temporal resolution) which is ideal for basin scale studies and multiple Landsat missions (30–100 m spatial and 16 days temporal) suitable for field level studies^14,17^. It is feasible now to setup operational monitoring systems across various scales from field to the basin and from districts to national and continental levels with daily, weekly, and biweekly temporal coverage using products derived from satellite data^10,16^.

Many approaches have been developed over the past four decades to estimate ET_a_ from remotely sensed satellite data^18^. One of the most commonly used, albeit a complex approach, is to use physically based Surface Energy Balance (SEB) models which require a combination of meteorological and satellite data as inputs^19–21^. There are many variants of SEB models including Atmosphere-Land Exchange Inverse (ALEXI)^22^, Mapping EvapoTranspiration at high Resolution with Internalized Calibration (METRIC)^23^, Surface Energy Balance Model for land (SEBAL)^19^, operational Simplified Surface Energy Balance (SSEBop)^24^, Simplified Surface Energy Balance Index (S-SEBI)^25^ etc, which have been successfully implemented at various scales from basin to global to encompass all scales in which SEB type models have been used. METRIC and SSEBop are successfully implemented in the United States and other countries reporting high accuracies with R^2^ reported ranging from 85 to 95% against field observations obtained from Lysimeter^26^. The SEBAL model used in this study is implemented in various studies that reported higher accuracies ranging from 85 to 96% respectively on daily to seasonal time scales^27^.

Implementing SEBAL model over a large area is often challenging due to complexity in data requirements, the need for internalized calibration, and geospatial big data processing^28^. Hence SEBAL applications have been mostly spatially limited to smaller geographical areas, e.g. Irrigation districts or sub-basins, and temporally to processing a handful of images in one growing season or a year^29^. However, with the development of cloud computing infrastructures there are community driven efforts such as OpenET aiming at increasing access to remote sensing based ET_a_ estimations irrespective of the models^30^. SEBAL implementation in Google Earth Engine called geeSEBAL automates the ET_a_ estimation using Landsat data^31^. The most important input data required from remote sensing is thermal radiation data from satellite sensors. Currently, thermal data acquired from Landsat and MODIS sensors are being used extensively for ET_a_ estimation at 30 m and 1 km respectively^26^. Note that the Landsat thermal bands are acquired at 100 m spatial resolution and resampled to 30 m by the data provider using cubic convolution method. However, the data obtained in the optical spectrum is often contaminated due to clouds leaving large gaps which makes it difficult to do monthly/seasonal aggregations. Statistical interpolation over spatial domain while applied to fill larger gaps results in over smoothing. Hence biophysical parameters following annual unimodal or multimodal cyclic patterns have been successfully gap-filled using a combination of temporal and spatial interpolations. Time series reconstruction and gap filling have been successfully implemented on daily NDVI using Fourier transform^32^, daily lake surface water temperature using the Harmonic ANalysis of Time Series (HANTS)^33^, daily Land Surface Temperature (LST) using weighted temporal averaging^34^ and Locally Weighted Regression (LWR) followed by spline based spatial interpolation^35^. Data fusion approach Spatial and Temporal Adaptive Reflectance Fusion Model (STARFM)^36,37^ and time series smoothening using Savitzky-Golay filter^38^, were used to reconstruct daily ET_a_ from combining Landsat ad MODIS spectral datasets. In this study, 30 m gap-filled monthly ET_a_ maps for two crop years from October 2013 to September 2015 over the large basin LUB in Iran was developed. An open source python based SEBAL implementation called PySEBAL library was used to compute ET_a_ from all the Landsat 7 and 8 images in the study years. A new approach based on temporal and spatial interpolation was developed to derive gap-filled monthly ET_a_ maps at high resolution over LUB in Iran.

## Study area

Lake Urmia Basin (LUB) is located in the North-West region of Iran with a total surface area of ~ 52,970 km^2^. Mean annual precipitation in the semi-arid basin is 350 mm and the mean annual temperature is 11.2 °C. The basin is an endorheic basin where no surface water outflow occurs. However, groundwater seepage and evaporation are the primary mechanisms of water loss from the basin. Lake Urmia is a terminal lake that is situated in this basin. All the runoff generated in the basin ends in this terminal lake through a network of rivers.

The past two decades have seen a staggering decline in the surface area of Lake Urmia. Once the world’s second-largest hypersaline lake with a surface area of nearly 5000 km^2^, the lake is now reduced to below 2000 km^2^. This phenomenon had significant impact on the livelihood of millions of people living in the basin and causing far reaching regional ecosystem and health consequences due to issues such as loss of biodiversity and salt storms. The large magnitude of the issues caused by the desiccation of Lake Urmia has triggered the scientific community to study the long-term land and water use in the basin thereby understanding the reasons behind such a phenomenon. Several terminal lakes such as Lake Chad in Africa^39^, the Aral Sea in central Asia^40^, and Lake Poopo in South America^41^, have been affected in the recent past and have witnessed significant shrinkages.

While the main reason behind the decline of the Lake Urmia remains a matter of dispute between scientists^42,43^, it is plausible that a combination of natural factors e.g. reduced precipitation^44^ and anthropogenic changes e.g. expansion of irrigated agriculture^45^ are the drivers behind the lake shrinkage. Whatever the causes may be, the only way out is to significantly curb water consumption in the basin. The main burden is on agriculture that at the moment claims more than 90% of renewable water resources and reduces the groundwater storage causing a significant drop in the water table in the aquifers^46^. Recognizing this, top-down strategies were drawn up by the government^47^ including targeting a 40% reduction of agricultural water use centered around reducing surface water allocation to agriculture accompanied by investing in a shift to drip irrigation and proposing a cropping pattern change. These interventions have been successful in bringing fragile stability in an otherwise catastrophic situation^48^. An important missing piece is, and has been, a detailed understanding of spatial and temporal variations in consumptive water use, i.e. ET_a_, in the basin especially in irrigated areas. Such information is crucial for pinpointing hotspots for water savings and making targeted investments in scaling solutions that are proven useful by means of measurements. Furthermore, given the fragility of the gains, continuous monitoring of the water use in the basin remains pivotal for any prosperous future outlook.

## Data

**Figure 1:**
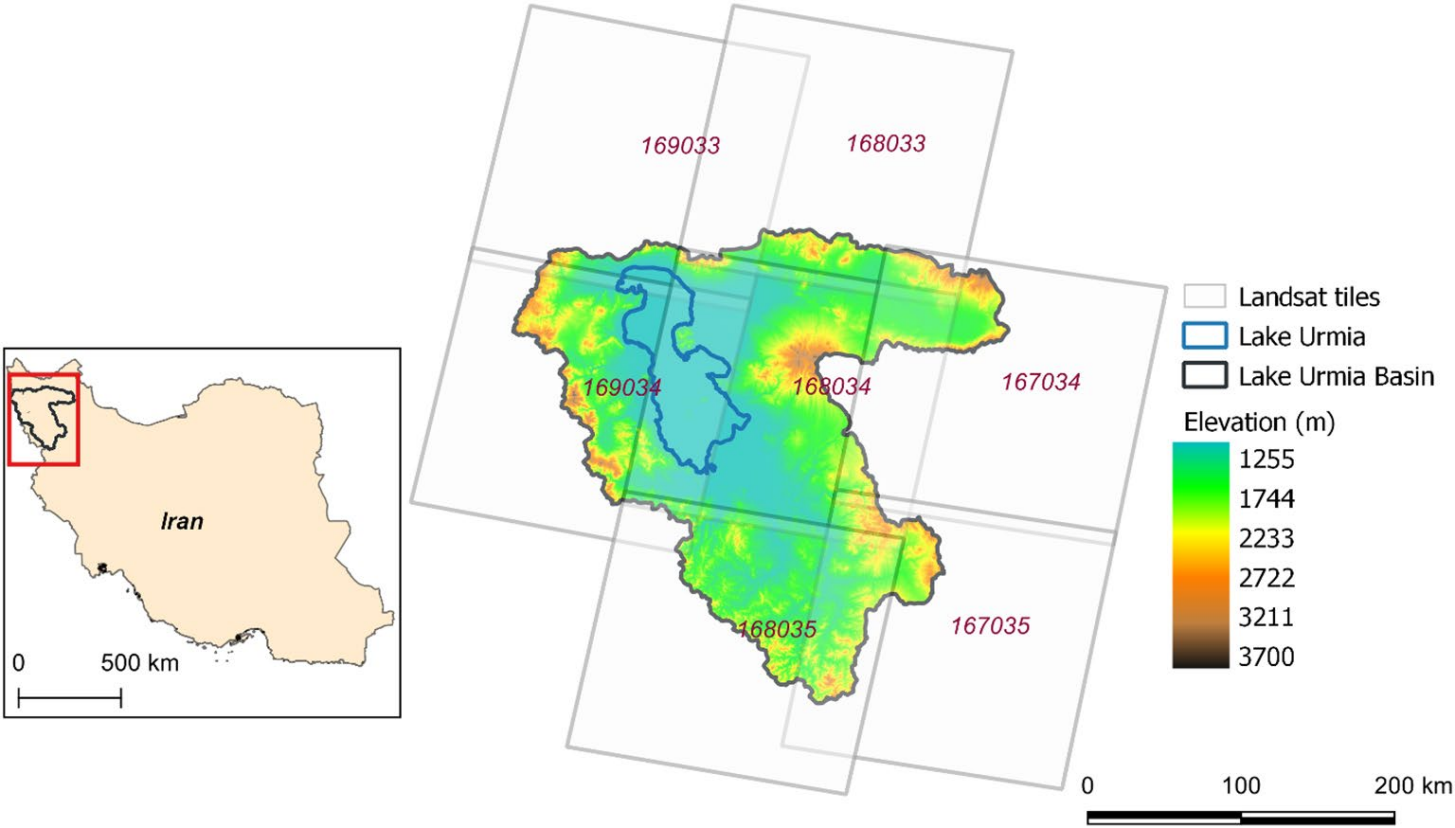
Study area—LUB boundary and Landsat tiles used for this study. The red box in the inset map shows the location of basin in Iran. Map created in QGIS 3.28.8 LTR software (https://www.qgis.org/).

**Figure 2:**
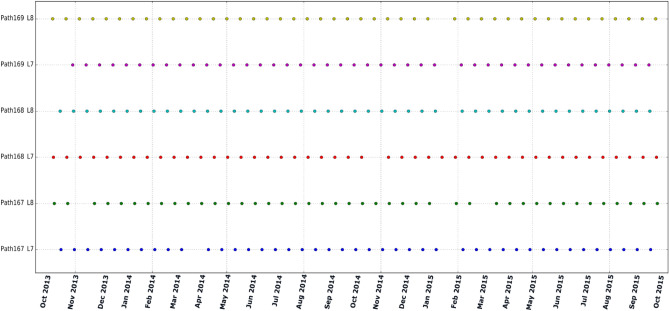
Available Landsat 7 and 8 over the study period from October 2013 to September 2015 separated by different paths covering LUB.

All the Landsat 7 and 8 images acquired between 1 October 2013 and 30 September 2015, were processed to estimate ET_a_ for the days of acquisition. This includes two tiles from path 167 and 169, and three tiles from path 168 (Fig. 1), and a total of 626 Landsat scenes were processed. Figure 2 shows the Landsat acquisitions in the study period used for applying SEBAL algorithm. The Landsat Collection 1 Level-1 data in Tier 1(T1) inventory were used for this study, acquired from Google cloud public storage using *gsutil* python library. The Landsat data provided in the T1 inventory are terrain corrected with well characterized radiometry and are inter-calibrated across different Landsat sensors^49,50^. For topography and elevation, Shuttle Radar Topography Mission (SRTM) data at 30 m spatial resolution obtained from the United States Geological Survey (USGS) repository were used.

Further, meteorological data at the time of satellite data acquisition (instantaneous) and 24 h average of the day of acquisition were required. The meteorological data required for SEBAL implementation are listed in Table 1. These data were extracted for the LUB from NASA Global Land Data Assimilation System (GLDAS v2.1), a global product developed by assimilating data from satellite and ground based observations^51^. GLDAS data is offered at 0.25 degree spatial resolution at 3 h interval. For comparison, Actual EvapoTranspiration and Interception (AETI—hereafter “ET_a_” for consistency) data at Level 1 (250 m resolution) obtained from Food and Agriculture Organisation (FAO) portal to monitor WAter Productivity through Open-access of Remotely sensed derived data (WaPOR) was used (url: https://wapor.apps.fao.org). FAO WaPOR is a remote sensing based spatial database on water productivity related indicators including ET_a_ which offer open data for African and near East countries. The ET_a_ data in WaPOR is computed using ETLook model based on the Penman–Monteith equation, which is modified to incorporate remote sensing input data and computes the combined rate of evaporation and transpiration^52^. It provides the highest available spatial resolution of 250 m for an operational open-access ET_a_ product at the continental scale^9^.

**Table 1:** Input meteorological data required for PySEBAL model.

Parameter	Symbols	Unit
Downward shortwave radiation	SWdown	W/m^2^
Wind speed	Ws	m/s
Air temperature	Tair	°C
Pressure	P	Mb
Relative humidity	Rh	%

## Methods

In this section, the implemented methodology grouped into (i) pre-processing of data, (ii) PySEBAL theory and (iii) the gapfilling approach are explained in detail.

### Pre-processing satellite and meteorological data

The acquired Landsat 7 and 8 data were pre-processed to compute cloud masked Top Of Atmosphere (TOA) reflectance bands at 30 m spatial resolution. The pre-processing included conversion from Digital Number (DN) to TOA reflectance, cloud removal using the Quality Assessment (QA) band and mosaicking the images acquired on the same day over a single path. The QA band contains 16-bit integers representing certain atmospheric conditions. All the bit combinations showing medium or high cloud confidence were used to create cloud masks. The pre-processing of meteorological data included the following steps (i) extracting the required variables as listed in Table 1 from the three hourly data, (ii) clipping the global data to the extent of LUB, (iii) converting the units of air temperature from Kelvin to °C, pressure from Pascal (Pa) to Millibar (mb), (iv) specific humidity in kg/kg to relative humidity in % and (v) extracting instantaneous and daily average meteorological variables. The instantaneous data corresponding to the Landsat acquisition time (7:30 A.M. UTC) was estimated by averaging the 6H and 9H UTC data, while all the 8 three hourly data in a day were averaged to estimate daily averages.

To efficiently process the Landsat data for a large basin such as LUB entire processing chain was implemented in a High Performance Computing (HPC) infrastructure. The entire processing chain was implemented by using multiple open source libraries, to deploy multi-core jobs like processing satellite data and to implement SEBAL model. All the spatial and temporal processing were performed in GRASS GIS 7.4.0 software^53^ which is open source under GNU General Public License (GPL).

**Figure 3:**
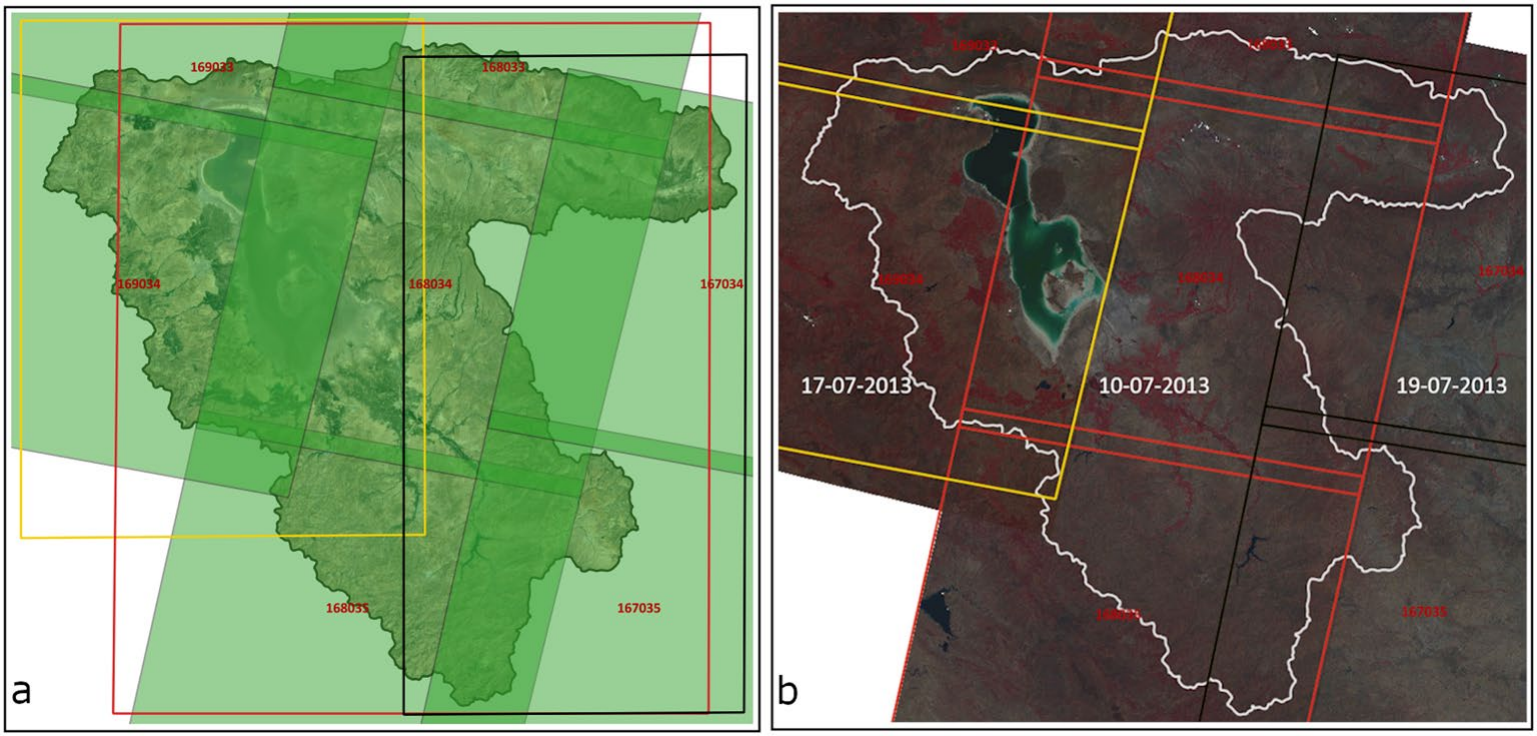
(**a**) Processing Units (PU) covering LUB; black rectangle—PU1 covering path 167; red rectangle—PU2 covering path 168; yellow rectangle—PU3 covering path 169, background satellite image source: Google Satellite API. (**b**) Mosaicked false colour composite from three paths acquired on three different dates. black rectangle—path 167; red rectangle—path 168; yellow rectangle—path 169, background satellite image source: Landsat 8. Maps created in QGIS 3.28.8 LTR software (https://www.qgis.org/).

The Landsat data processing for each path (167, 168 and 169) were performed in different nodes in parallel. This approach resulted in substantial reduction of the processing time. The data processing was divided into three Processing Units (PU) based on the Landsat path and row. Since the adjacent tiles in the same path have same acquisition date, we chose each path as a processing unit. Thus, data for each path were processed in parallel in the HPC. Figure 3 shows the bounding box of the three defined PUs for LUB (see Fig. 1 for complete view of Landsat tiles covering LUB). The PU’s are designed to incorporate minimum overlapping “rectangular” area between the respective Landsat path and the LUB. While processing in each PU, only those pixels inside the LUB boundary were considered further optimizing the efficiency in computing over larger area.

### SEBAL implementation in Python—PySEBAL

SEBAL work on the basic principle of solving the energy balance model as shown in Eq. (1) where latent heat flux (LE), the energy consumed through evapotranspiration, is computed as residual energy from net radiation (Rn), ground heat flux (G) and sensible heat flux (H).

1 $$LE = Rn - G - H$$

Instantaneous ET_a_ (mm/day) is then calculated using Eq. (2) where LE is divided by the latent heat of vaporization (L) and the water density (ρ_w_).

2 $$ET_{a} = \frac{LE}{{L*\rho_{w} }}$$

PySEBAL is a python library to implement SEBAL using spectral reflectance values from satellite data, climatic parameters and topography as input to estimate the surface energy balance components. The outputs include parameters related to vegetation, energy balance, biomass, evapotranspiration, and water productivity. Currently, PySEBAL supports data from MODIS, Landsat, and Proba-V satellite sensors which facilitate the production of daily and seasonal ET_a_ maps. PySEBAL have been used for estimating water use in agriculture by^54–56^.

**Figure 4:**
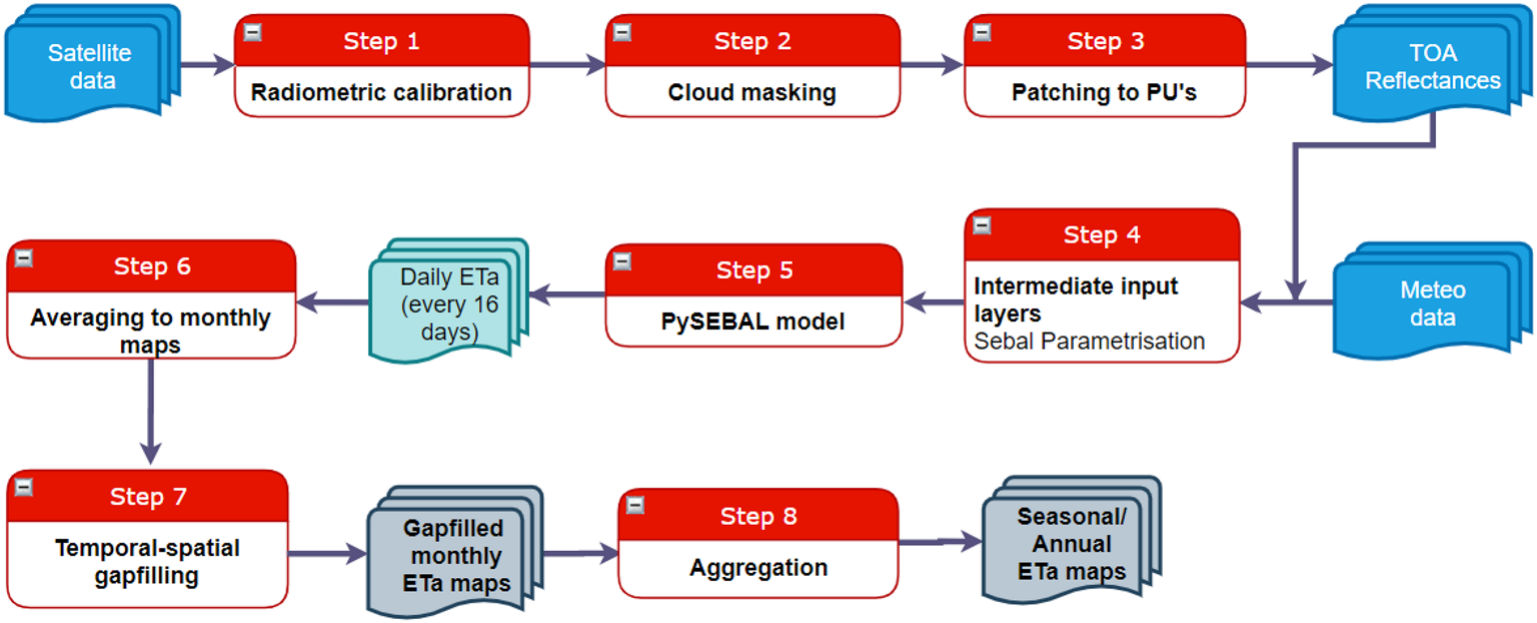
Work flow diagram showing all the steps included in the method including input data, PySEBAL model and gap filling process.

Figure 4 shows the workflow of computing seasonal ET_a_ using PySEBAL.The steps 1 and 2 in the workflow diagram cover the pre-processing of Landsat data and cloud masking as explained in the previous section. In step 3, each spectral band of Landsat are patched/mosaicked to respective PU’s. Step 4 is to create intermediate input layers that are required for closing energy balance equation which includes Normalized Difference Vegetation Index (NDVI), Soil-Adjusted Vegetation Index (SAVI), Leaf Area Index (LAI) and Surface Albedo (*α*) from Landsat spectral data. Step 5 computes the energy balance components Rn, G, and H as shown in Eq. (1).

The equations used behind computing the major energy balance components in SEBAL is well explained in^19,20,57^. The computation of these components in PySEBAL is demonstrated in^54,55^. The net radiation flux (Rn) represents the actual radiant energy available at the surface. Rn (W/m^2^) is the difference between all incoming radiant fluxes and outgoing radiant fluxes using Eq. (3)^23^.

3 $$R_{n} = \left[ {R_{{{\text{S}} \downarrow }} + R_{{{\text{L}} \downarrow }} } \right] - \left[ {\alpha R_{S \downarrow } + R_{L \uparrow } + \left( {1 - \varepsilon_{0} } \right)R_{L \downarrow } )} \right]$$

where ${\text{R}}_{{{\text{S}} \downarrow }}$ is the incoming solar radiation in W/m^2^, ${\text{R}}_{{{\text{L}} \downarrow }}$ and $R_{L \uparrow }$ is the incoming and outgoing longwave radiation respectively in W/m^2^, *α* is surface albedo (dimensionless) and $\varepsilon_{0}$ is the thermal emissivity in W/m^2^.

Ground heat flux (G) is the rate of heat storage into the soil and vegetation due to conduction. In PySEBAL, G in W/m^2^ is computed using Eq. (4) as function of surface temperature (T_s_), surface albedo (*α*), and NDVI^19^ after applying a water mask.

4 $$G = T_{s} \left( {0.0038 + 0.0074\alpha } \right)\left( {1 - 0.98 NDVI^{4} } \right)$$

Sensible heat flux (H) is the rate of heat loss to the air by convection and conduction, due to a temperature difference and it is computed using the Eq. (5).

5 $$H = \frac{{\rho_{air} C_{p} dT}}{{r_{ah} }}$$

where $\rho_{air}$ is air density, *C*_*p*_ is the specific heat of air at constant pressure, and dT is near-surface air temperature difference for each pixel and r_ah_ is the aerodynamic resistance. As the actual absolute values for air temperatures above each pixel are unknown, dT is computed by assuming a linear relationship between dT and altitude corrected surface temperature. To define the slope and offset of this linear model, SEBAL uses the two “anchor” pixels where a value for H can be reliably estimated. For each satellite image, these “anchor” pixels are selected by picking hot and cold pixels over the driest surface in the satellite scene and wettest surface such as water body or irrigated area respectively. The PySEBAL library facilitates automation of the entire process including the estimation of the hot and cold pixels from a satellite image. The cold pixels are usually selected from water bodies or crop areas with well-developed vegetation, whereas hot pixels are selected from completely dry soil surfaces. In PySEBAL, cold pixels are automatically identified based on thresholds (2nd and 5th percentiles) applied to distribution of surface temperature estimated from the Landsat thermal data, while hot pixels are identified from the distribution of NDVI, where 1st and 3rd percentiles are used as thresholds. The instantaneous latent heat flux (LE) and Evaporative Fraction (EF) at the time of satellite data acquisition is then computed from the energy balance components. The instantaneous EF is converted to the daily evaporative fraction (EF_24_) by incorporating an advection factor, which takes into consideration the vapor pressure deficit and accounts for the rise in ET during the afternoon period^55^. The daily ET_a_ is then computed by multiplying EF_24_ with 24-h net radiation where negligible ground heat flux over 24 h is avoided. The PySEBAL is capable of processing multiple satellite scenes in a single run. The optimal way is to set up and run PySEBAL for an entire season. Steps 6 to 8 in Fig. 4 represents the gap filling procedure which is explained in detail in the next section.

### Gap filling of ET_a_ maps

For every month, Landsat has 4 observations, two from Landsat 7 and two from Landsat 8, 16 days being re-visit time of both satellites. It is possible to have up to 4 observations per month per pixel, but often this is not the case due to cloud cover in the region especially during the winter season. All available scenes from Landsat 7 and 8 are incorporated in the processing, in order to increase the probability of having maximum valid pixels over a year. First step in the gap-filling procedure (step 6 in Fig. 4) is to patch all the ET_a_ maps by averaging them per month. The monthly ET_a_ maps were then converted from mm/day to mm/month by multiplying each map by number of days in the respective month. The remaining gaps were then filled using a temporal and spatial interpolation.

A temporal interpolation based on LWR was applied to the monthly ET_a_ maps to reconstruct missing values and identify outliers^35,58^. For each time series observation (pixel) in the map, a polynomial model of second order is computed using a set of neighboring pixels in the temporal dimension. Distance based weight is applied to the values in such a way that the observation farther away in time gets lower weights. All the observations in the time series were interpolated, as long as there were enough non-null observations. To keep the interpolated values within the seasonal limits, a maximum gap of 3 observations in the time series were interpolated, otherwise retained as NULL. The weight function used for LWR was Tricube which determines the influence of neighboring values in time to the current observation. High and low outliers in model fitting were ignored and extrapolation was avoided. The LWR addon in GRASS GIS 7.4.0 (*r.series.lwr*) was used to implement temporal interpolation.

Due to the insufficient valid observations in the time series meeting the LWR conditions there were remaining gaps in the monthly ET_a_ maps (less than 10% of the surface area). These gaps were then reconstructed using bicubic spline spatial interpolation. This step is applied only to the NULL pixels using the neighboring valid pixels, which means the observations and the temporally interpolated values were kept unchanged. Bicubic spline interpolation is a 2-dimensional approach to the linear spline. In this case, a minimum of neighboring 16 valid pixels are used to interpolate the null pixels using a cubic function. For each pixel, the interpolation takes into consider the function itself, the gradients determined by one-dimensional splines and the cross derivatives. The values of the function and the derivatives are reproduced exactly at the pixels and they change continuously with the moving window crossing one pixel to another. The bicubic approach ensures the continuation of derivatives to the adjacent grids thereby reducing the artefacts. The interpolation module in GRASS GIS 7.4.0 (*r.fillnulls*) was used to implement spatial interpolation in each monthly map.

After the gap-filling process applied to all the monthly maps from October 2013 to September 2015, the monthly maps were aggregated to create annual maps (mm/year) for the years 2013/2014 and 2014/2015. To assess the performance of this gap-filling approach, the gap-filled ET_a_ maps were compared with data obtained from FAO WaPOR over the LUB. The data from WaPOR were resampled from 250 to 30 m using nearest neighbor algorithm to match the gap-filled ET_a_ maps. Further to check the spatial and temporal consistency of the gap-filled maps, ET_a_ dynamics over different agricultural land use types and its response to bio-physical parameters like NDVI were investigated. The monthly ET_a_ estimates were compared to aggregated monthly precipitation obtained from rainfall stations in the LUB to understand the influence of irrigation in driving the water use in the basin.

## Results

Gap filled monthly ET_a_ maps (24 maps) at 30 m resolution for LUB were developed and analyzed. To demonstrate the different steps of gap-filling process, results obtained after each step is explained over the month of October 2014 as an example. Table 2 lists the available Landsat data for each PU (path) for October 2014 (see Fig. 2 for availability of Landsat 7 and 8 over the study period). There were total 10 Landsat scenes available in October 2014 (Four Landsat 7 and Six Landsat 8).

**Table 2:** List of Landsat 7 and 8 data available and processed for the month of October 2014.

	Landsat 7	Landsat 8
PU1 (path 167)	02-10-2014	10-10-2014
	18-10-2014	26-10-2014
PU2 (path 168)	09-10-2014	01-10-2014
		17-10-2014
PU3 (path 169)	16-10-2014	08-10-2014
		24-10-2014

**Figure 5:**
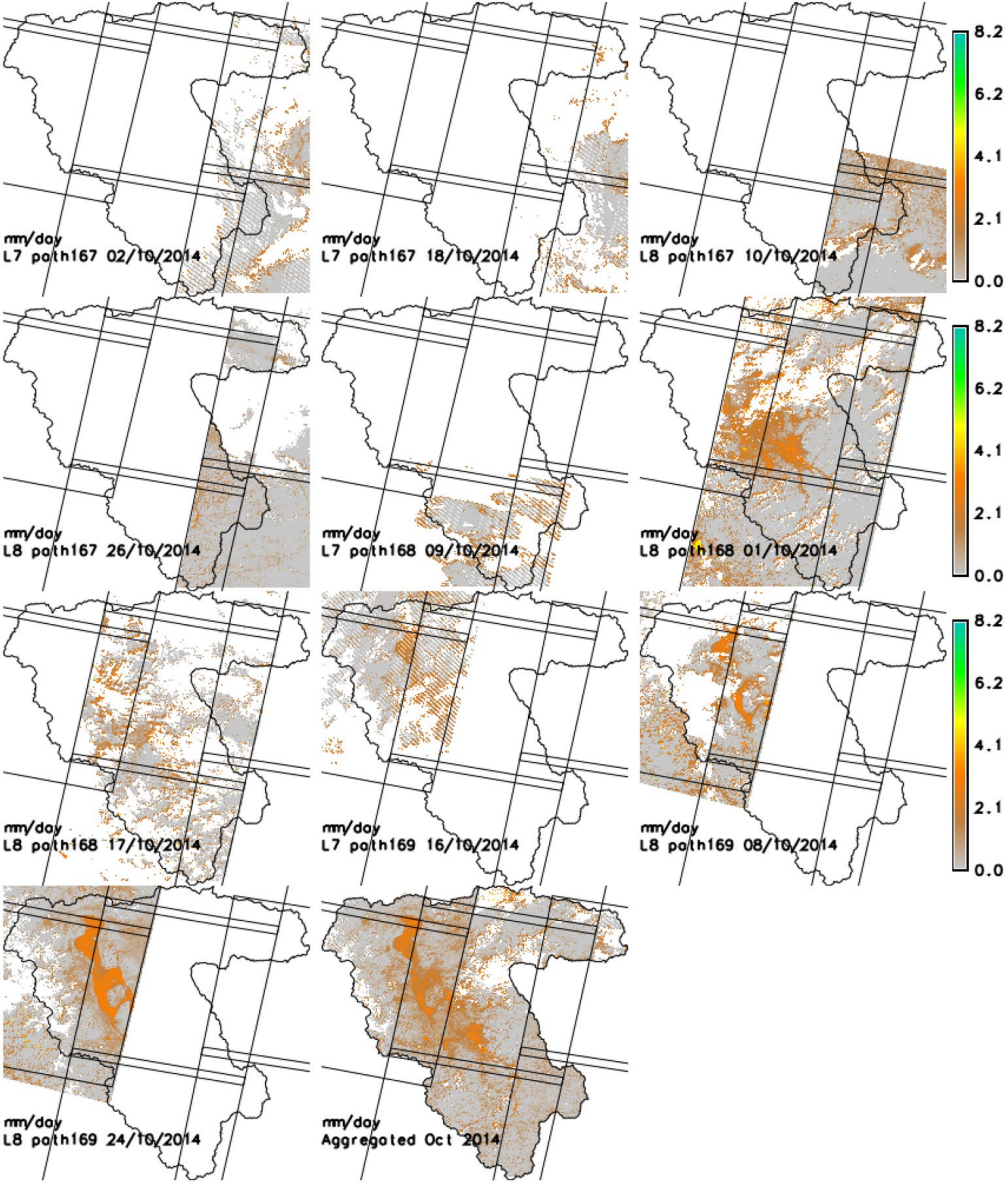
Individual ETa maps from October 2014; the last block shows the aggregated average ETa map for the same month. Unit is mm/day. Map created in GRASS GIS 7.8.4 software (https://grass.osgeo.org/).

Figure 5 shows the individual 10 ET_a_ maps computed using PySEBAL from Landsat acquisitions of October 2014 for the three PU’s. These 10 maps were then aggregated by averaging and clipped to LUB (see the last map of Fig. 5).

**Figure 6:**
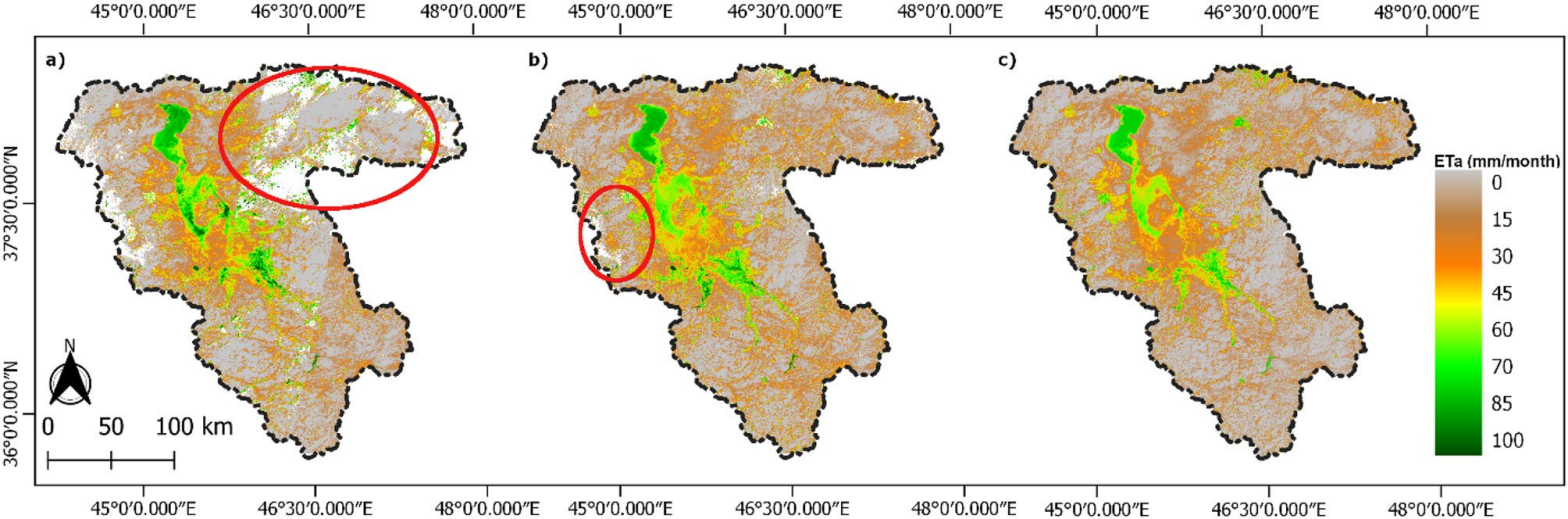
Monthly ET_a_ map for the month October 2014 (**a**) after aggregation of individual ET_a_ maps, (**b**) after LWR interpolation, (**c**) gap-filled ETa monthly map after spatial interpolation; red circles shows the gaps due to clouds. Maps created in QGIS 3.28.8 LTR software (https://www.qgis.org/).

**Figure 7:**
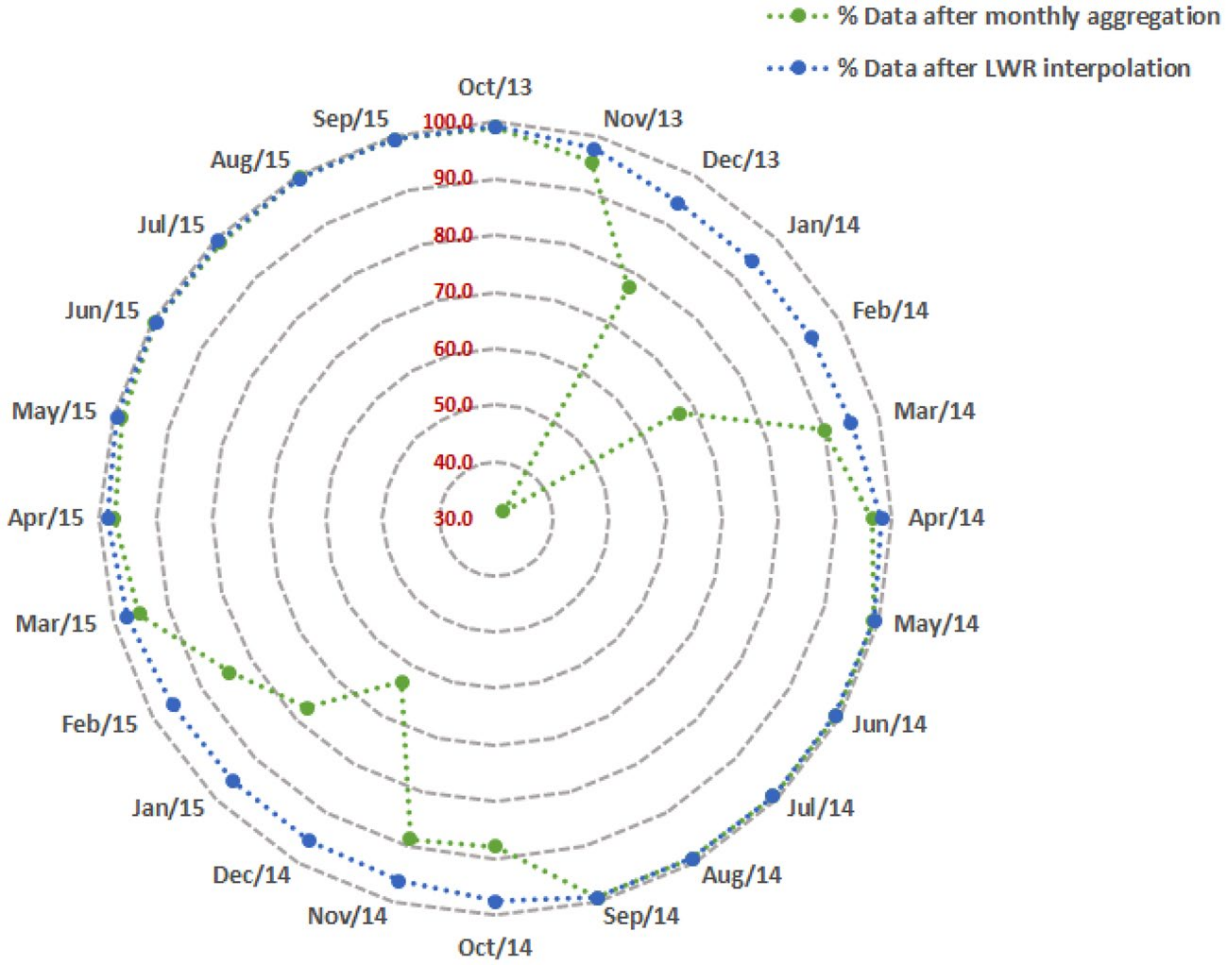
Percentage of valid monthly observations after monthly mean aggregation and LWR interpolation.

Figure 6 shows the gap filled ET_a_ map of October 2014 (mm/month) before and after the gap filling. Similarly, for each month from October 2013 to September 2015, there were a maximum of 12 observations per month for the basin and computed gap-filled monthly ET_a_ maps at 30 m spatial resolution. The variation of gaps in the ET_a_ maps over LUB after each step in the gap filling procedure was analyzed to understand the pattern of reconstructing the maps. The results show that, by combining Landsat 7 and 8 data substantially reduced the cloud coverage when aggregated to monthly maps. After monthly aggregation by averaging of all the individual ET_a_ maps the maximum cloud cover was 70% over entire LUB in January 2014 followed by 42% cloud cover in January 2015. After LWR interpolation majority of the gaps in the winter months were statistically filled to 90%, while all the summer months were completely gap-filled. Figure 7 shows the variation in gaps due to cloud cover over 24 months before and after the gap filling steps.

**Figure 8:**
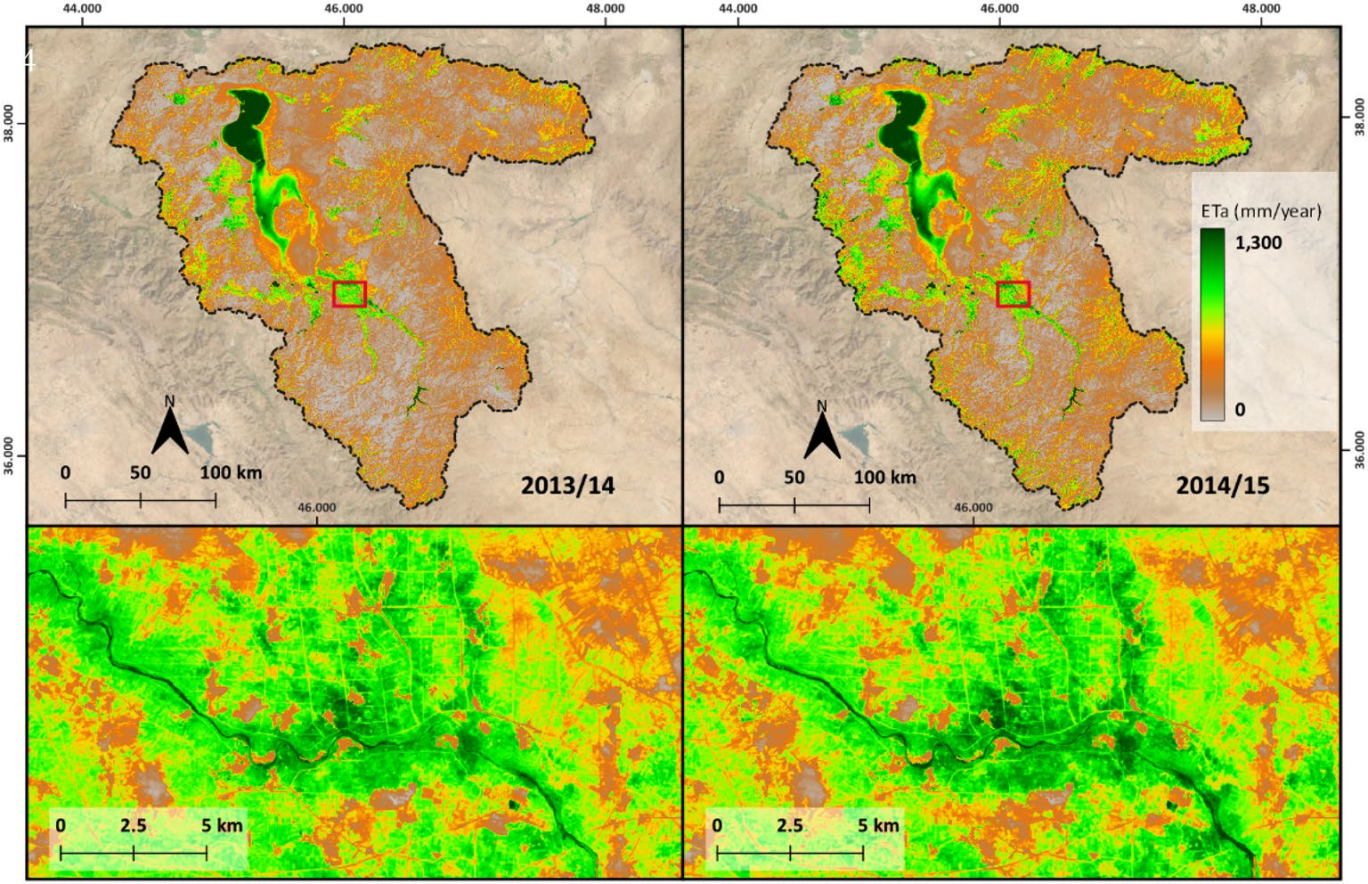
Top: Gap-filled annual ET_a_ maps of LUB for the years 2013/14 and 2014/15, background satellite image source: Sentinel-2 cloudless 2016 by EOX IT Services GmbH; Bottom: zoomed into an irrigated area in Miandoab irrigation scheme, this area is indicated in red box above. Map created in QGIS 3.28.8 LTR software (https://www.qgis.org/).

**Figure 9:**
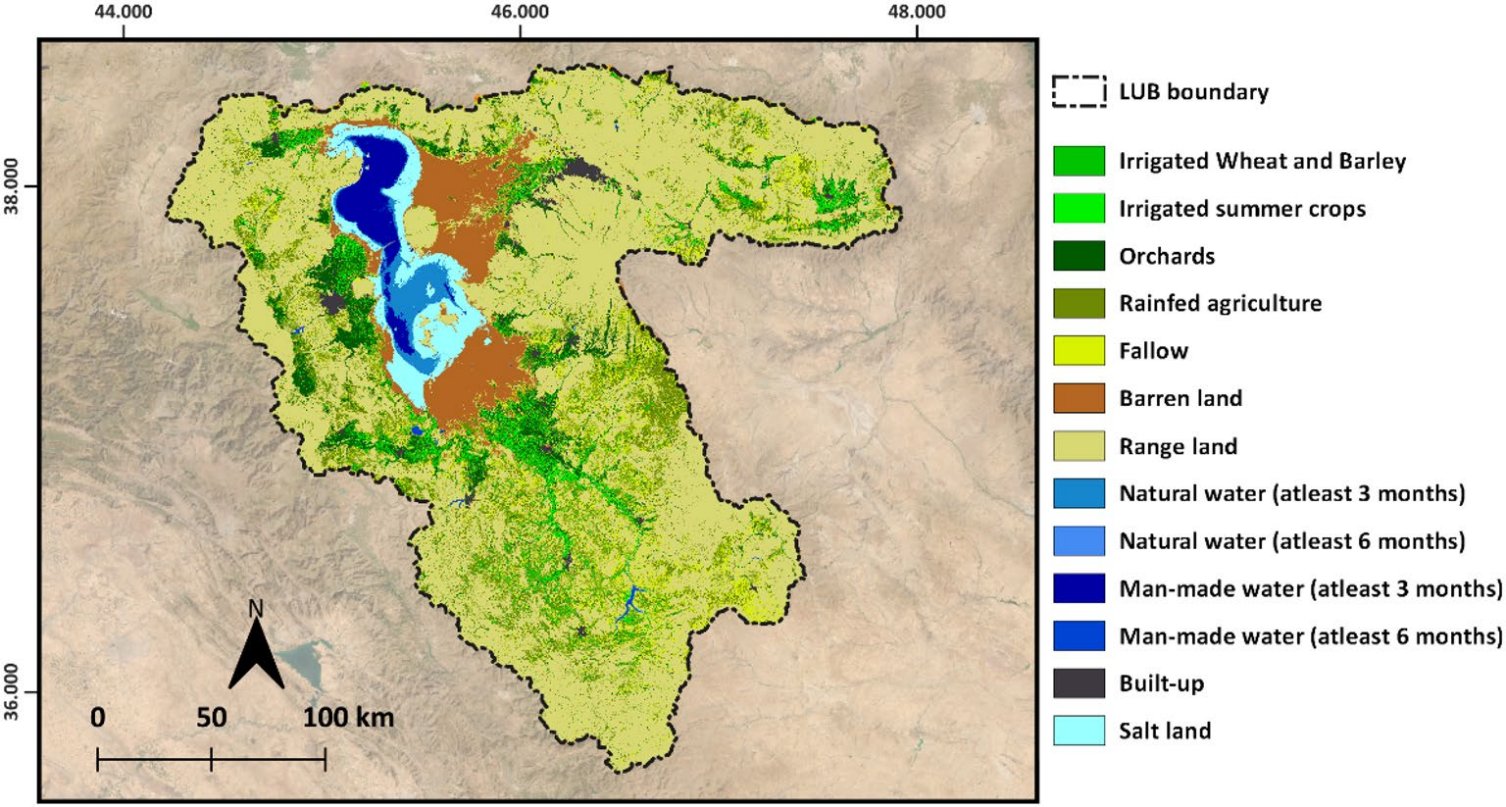
Land use map of Lake Urmia Basin (source: Tarbiat Modares University, Iran). Background satellite image source: Sentinel-2 cloudless 2016 by EOX IT Services GmbH. Map created in QGIS 3.28.8 LTR software (https://www.qgis.org/).

Figure 8 shows the final gap-filled annual ET_a_ maps for two crop years over LUB. The annual average ET_a_ estimated from the gap-filled monthly maps was 302 mm and 346 mm in the crop years of 2013/2014 and 2014/2015 respectively. The annual rainfall reported was 306 mm and 368 mm in the crop years 2013/2014 and 2014/2015 respectively. Correlation analysis between monthly gap filled ET_a_ and FAO WaPOR reported a coefficient of determination (R^2^) of 0.93, Root Mean Square Deviation (RMSD) of 9 mm/month, Mean Absolute Deviation (MAD) of 7 mm/month and Mean Absolute Percentage Deviation (MAPD) of 36% over agriculture area. The land use map used to select agriculture area for correlation analysis is shown in Fig. 9.

**Figure 10:**
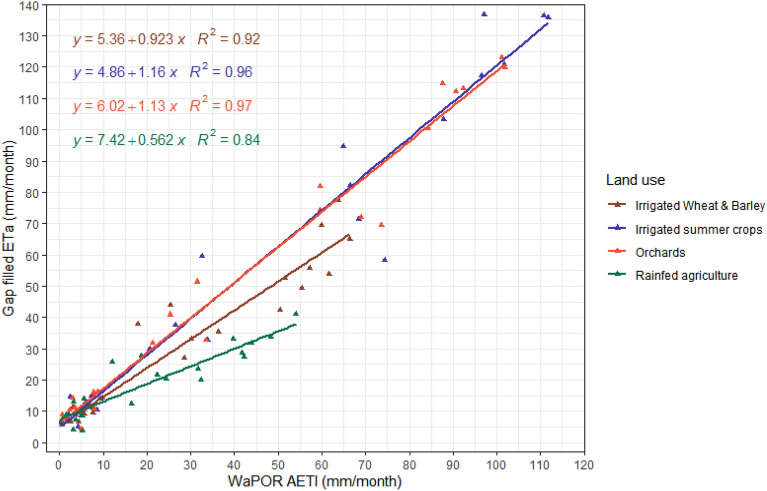
Scatter plot between monthly gap filled ETa and FAO WaPOR AETI with best fitting linear regression lines over four agricultural land use types.

**Figure 11:**
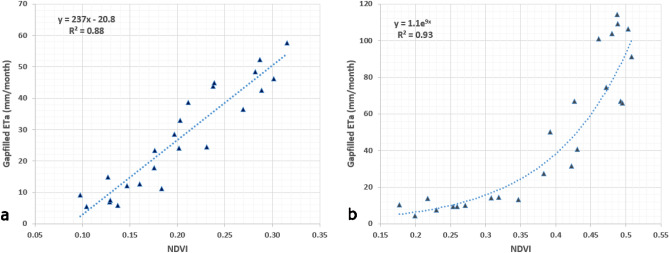
Scatterplot between monthly NDVI and ETa (**a**) over the entire basin except water bodies; (**b**) over the irrigated wheat and barley, irrigated summer crops and orchards.

Comparison statistics between gap filled ET_a_ and FAO WaPOR data over different agriculture land use types—irrigated wheat and barley, irrigated summer crops, orchards and rainfed are listed in Table 3. The scatter plot between gap filled ET_a_ and FAO WaPOR data is shown in Fig. 10 fitting the linear models over four agriculture land use types. Correlation of these two datasets over orchards was reported to be the highest with R^2^-0.97. RMSD and MAD over orchards were 6 and 4 mm/month respectively. For irrigated wheat and barley and irrigated summer cropped area the R^2^ reported were 0.91 and 0.95 respectively showing very high correlation between the datasets. While the RMSD reported over these land use types were 7 and 8 mm/month, estimated MAD was 5 mm/month. Correlation over rainfed area was reported to be lowest with R^2^—0.84, RMSD—7 mm/month and MAD – 5 mm/month. MAPD ranged from 30 to 46% for irrigated wheat and barley and rainfed area respectively. Table 3 also lists the comparison statistics between ET_a_ before gap filling and WaPOR data. The results shows that gap filling didn’t improve the ET values over irrigated wheat and Barley (winter season) and rainfed, while it improved substantially over the summer irrigated crops and orchards. In case of winter irrigated crops and rainfed, the RMSD increased from 4 to 6 mm/month. For orchards and irrigated summer crops the deviation between gapfilled ET_a_ and WaPOR reduced substantially. RMSD improved from 25 to 8 mm/month for irrigated summer crops, while it improved from 19 to 6 over orchards. Further the response of gap-filled ET_a_ to NDVI showed a linear correlation with R^2^ of 0.88 and an exponential fit with R^2^ 0.93 over irrigated wheat and barley, irrigated summer crops and orchards. Figure 11 shows the scatter plot between gap-filled ET_a_ and NDVI with fitted regression models.

**Table 3:** Comparison statistics reported comparing monthly before and after gap filled ETa and FAO WaPOR data over two study years.

	R^2^		RMSD (mm/month)		MAD (mm/month)		MAPD (%)	
	Before gapfilling	After gapfilling	Before gapfilling	After gapfilling	Before gapfilling	After gapfilling	Before gapfilling	After gapfilling
Irrigated Wheat and Barley	0.97	0.92	4	7	2	5	13	30
Irrigated Summer crops	0.72	0.96	25	8	18	5	82	35
Orchards	0.8	0.97	19	6	14	4	76	34
Rainfed	0.8	0.8	4	7	3	5	39	46

**Figure 12:**
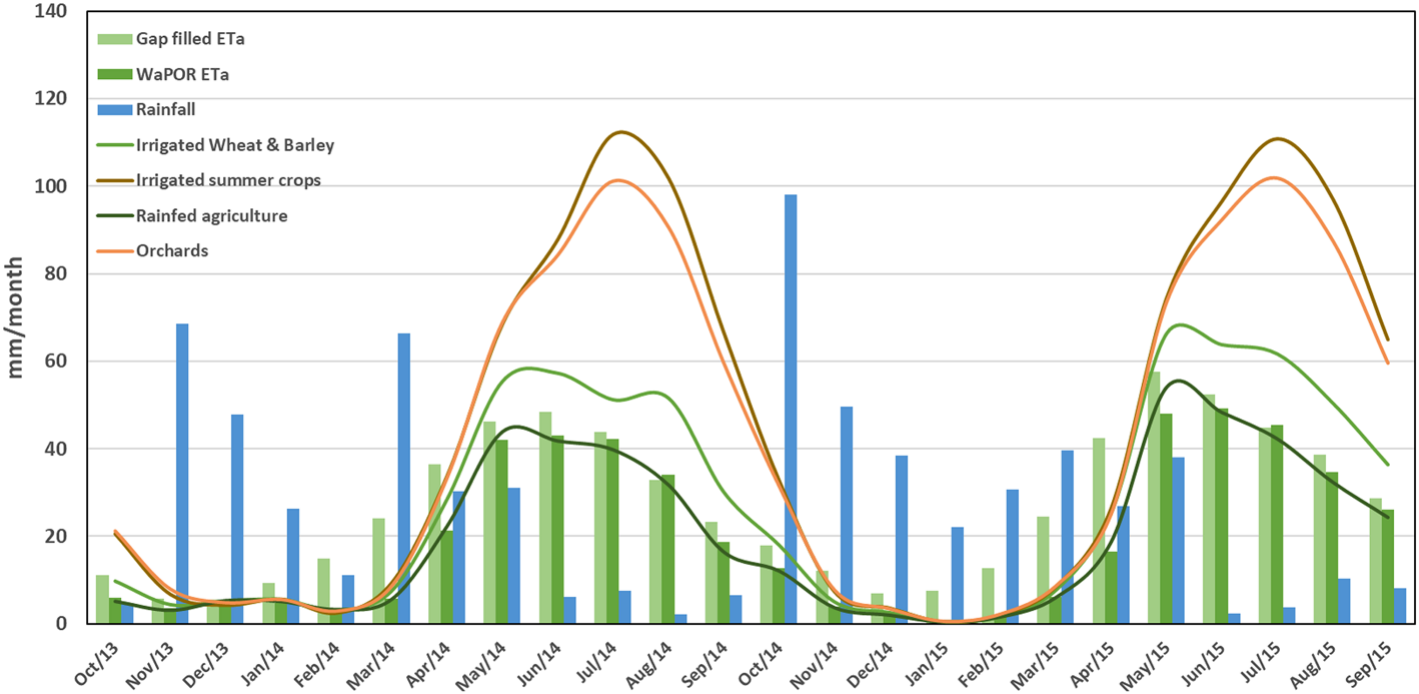
Monthly P and ET variations in LUB.

The monthly ET_a_ and Precipitation (P) were compared to analyze the underlying drivers of the seasonal trend of water use over different land use types. Figure 12 shows the temporal variation of monthly ET_a_ (gapfilled and WaPOR) and P over the LUB and also the monthly gapfilled ET_a_ over the agricultural land use types for the two crop years from October 2013 to September 2015. The rainfall during the second cropping year (mean rainfall of 368 mm) was greater than the previous year (mean rainfall of 306 mm). It also reflects in the estimated ET_a_ with a higher average reported in the second year (346 mm) than first year (302 mm). There were extremely high rainfall events reported in the month of October 2014 (~ 95 mm) which also triggered the higher ET_a_ range in the second cropping year.

**Figure 13:**
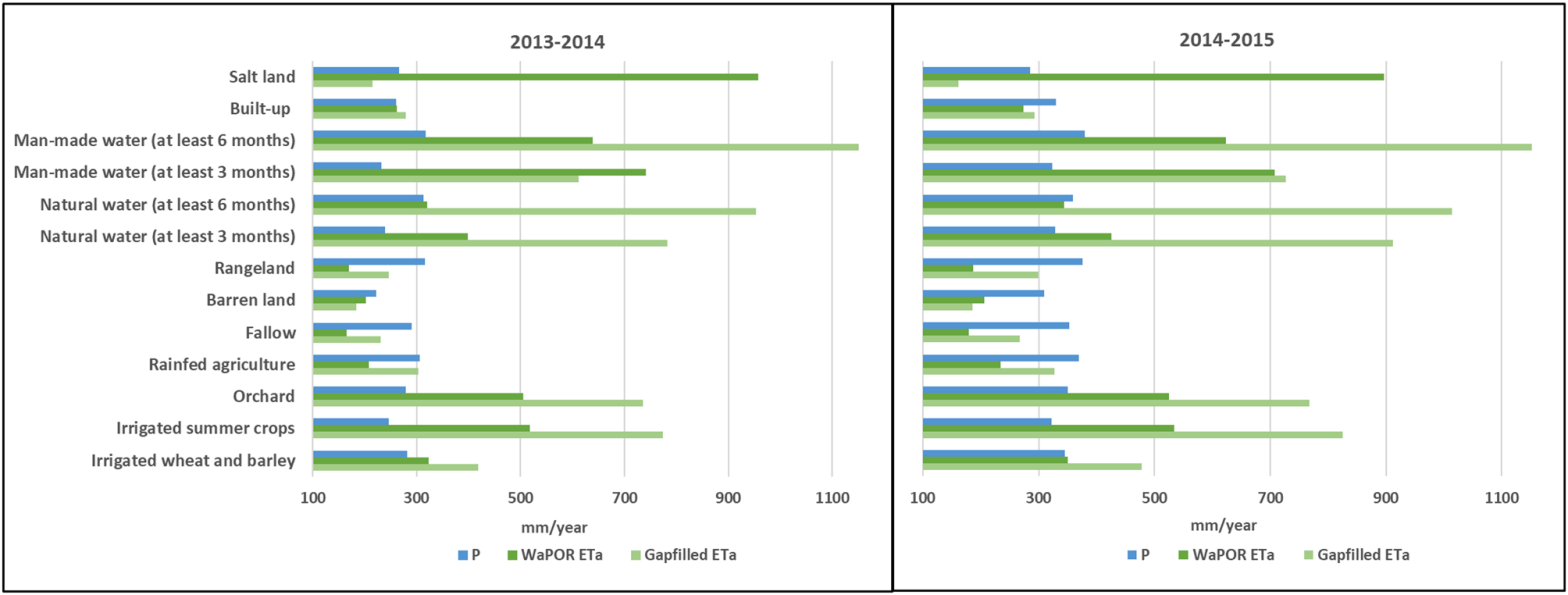
Annual P and ET_a_ over different land use classes for two crop years left—2013/14 and right—2014/15.

The land use based ET_a_ and P estimates over the study period are illustrated in Fig. 13. The higher ET_a_ values reported during the summer months from April to September in both cropping years were contributed by the irrigation events and evaporation from water bodies. To understand further the land use based dynamics of ET_a_ and P, mean ET_a_ based on land use types were extracted. Over the two crop years highest ET_a_ (except water bodies) of 800 mm was reported for the irrigated summer crops land use type followed by orchards with mean ET_a_ of 751 mm. The lowest average ET_a_ of 199 mm was reported for the barren land. Agriculture related land use types were reported to have highest ET_a_ over the study period as expected.

## Discussion

Quantifying water use by agriculture land use types is of utmost importance to take actionable measures to avoid overuse of resources. The study demonstrates the use of publicly available earth observation satellite data acquired by Landsat 7 and 8 to monitor water use over a large basin at different temporal scales. The approach implemented over a large basin covered by 7 Landsat tiles could efficiently reconstruct the gaps in data due to clouds and stripes on Landsat 7 due to the scan line corrector failure. The parallelization of the process with the implementation of processing units covering tiles of same path substantially reduced the processing time.

The inclusion of data from both Landsat 7 and 8 increased the availability of observations in space–time domain thus reducing the gaps after step 6 of monthly aggregation in methodology (Fig. 4). This is reflected in the cloud dynamics after monthly aggregation (Fig. 7) where most of the months other than January have good observations for more than 60% of the basin. The possibility to use both Landsat 7 and 8 in PySEBAL and the inter-calibration of reflectance values by the Landsat data provider makes it possible to combine data from Landsat 7 and 8 (USGS, 2019).

The conversion of monthly ET_a_ maps in mm/day to mm/month by multiplying number of days in respective month assumes that the ET_a_ values are constant throughout that month. The ET_a_ can vary significantly within a day or a month depending on the land cover type. This might influence the accuracy of ET_a_ measured using sparse observations from Landsat. The LWR is able to capture the short-term fluctuations in the time series compared to approaches like harmonic analysis^35^. This is useful while planning interventions based on short term water use of crops within a season. LWR in this case could fill the majority of the gaps even after applying stricter constraints especially in the number of samples per regression (limited to 3), thus avoiding overfitting of the predictions. After the time series smoothening using LWR, the gaps were reduced to less than 10% in all the months. For a diverse terrain like LUB, the time series reconstruction at high resolution allows the capturing of ET_a_ temporal dynamics for different land use types as demonstrated in this study.

The variation of monthly ET_a_ over agriculture land use types—irrigated wheat and barley, irrigated summer crops, orchards and rainfed showed distinctive seasonal variations in response to the rainfall and cropping patterns. For the irrigated wheat and barley, the ET_a_ is highest in April and declines gradually by late June. This match with the cropping calendar for winter wheat and barley in the region. For orchards, ET_a_ showed peak values during summer months (May to Aug) and lower values were observed from September onwards and in the winter season. As expected, the water consumption by orchards were much higher than irrigated wheat and barley and lower than Irrigated summer crops^59^. This could be attributed to inclusion of young orchards in this land use class and also the fact that orchards besides irrigation schemes are present in the valleys in the vicinity of surface streams throughout the basin where irrigation is not as intensive as in the schemes.

The non–irrigated land use types showed much lower ET_a_ compared to the irrigated classes. The peak ET_a_ was observed in months of May at an average value of about 2 mm/day for rainfed crops and 1.5 mm/day for rangelands. Similar study reported an average ET_a_ of 736 ± 42 mm for over the irrigated agriculture in the basin in 2014, the average ET_a_ for irrigated agriculture in our assessment was 696 mm^59^. It demonstrates the usability of such maps in comparison with rainfall to give us an overall account on critical water availability versus water consumption over managed landscapes and water deficit. The linear correlation between ETa and NDVI over the entire basin as shown in Fig. 11a is influenced by the rangeland pixels which forms major land cover in the basin (64% of total area). While the scatterplot as shown in Fig. 11b over the irrigated area which is a managed land cover type shows an exponential relation representing the yield response to water applied for the irrigated crops.

The comparative analysis between newly developed monthly gap-filled ET_a_ and AETI from FAO WaPOR dataset reported R^2^ greater than 0.9 for irrigated wheat and barley and summer crops, while a R^2^ of 0.84 was reported in rainfed areas. The FAO WaPOR level 1 AETI data used in this study was developed using 8-day MODIS products at 250 m and the difference in spatial resolution could be a contributing factor for higher RMSD of 7–9 mm/month and MAPD greater than 30%. The correlation was worsened after gapfilling over the irrigated wheat and barley potentially due to multiple factors like higher percentage of gaps due to cloud cover during the winter months (see Fig. 7), undetected cloud and cloud shadow pixels, coarse spatial resolution and the difference in interpolation techniques used in WaPOR database. WaPOR level 1 data is reported to be underestimating the ET_a_ values due to the coarse resolution of input land surface temperature data (1 km) from MODIS sensor which is used to derive moisture stress and thus affecting the spatial variation^60^. A recent study evaluating the consistency between different levels of WaPOR data found higher correlation between level 1 AETI and the field observations over Zankalon irrigated area in Egypt^61^.

Remote sensing based ET_a_ mapping in many studies is performed by establishing direct empirical relation between NDVI and crop coefficient (Kc) instead of solving surface energy balance equitation^62,63^. Hence, investigating relation between aggregated ET_a_ values derived from PySEBAL and the NDVI values directly acquired from satellites provide insights into the quality of derived ET_a_. It is expected that ET_a_ and NDVI show a general correlation which could be stronger in land use classes that typically have higher vegetation growth such as orchards and summer crops. The linear relationship between ET_a_ and NDVI over all the land use types demonstrates vegetation growth with increasing availability of water. The vegetation indices from multi-spectral satellite data can well describe the vegetation growth and thereby can explain the water use trend. Hence the expected response of ET_a_ to the vegetation growth as indicated by the time series of NDVI would give us quality assurance of the developed monthly ET_a_ maps.

For irrigated landscapes such as orchards and irrigated summer crops, seasonal prevalence of evaporation and transpiration play key role in formulating the relationship between ET_a_ and NDVI. The models revealed that the vegetation growth in these land use types are not limited to availability of water. During the winter months and beginning of the growing season, a rapid response of vegetation growth with lower ET_a_ values with prevalent contribution from evaporation was observed. After the vegetation growth is matured (corresponding to NDVI value of 0.43 and ET_a_ of 60 mm—see Fig. 11), the response becomes slow which requires much more water to attain rest of the growth which are provided by irrigation.

The newly developed gap-filling approach can be used to monitor the water use estimation by different land use types in a larger basin like LUB. In LUB, periodic monitoring of land and water use will support the water management interventions to be taken in order to revive the Lake Urmia. This data can be further used for assessing water productivity, irrigation performance and computing water accounts at different scales from field to basin^12^. Validating the remote sensing based ET_a_ data remains a challenge due to lack of wide network of in-situ flux data. Hence, the alternative is to perform inter-comparison with similar products and analyze the spatio-temporal trends of ET_a_ in different land use types. There are multiple projects being carried out to improve this and to develop robust protocols to assess remote sensing based ET_a_ products (see WaterPIP project; url: http://waterpip.un-ihe.org/).

## Conclusion

The increasing availability of open access satellite data and new advances in remote sensing techniques are paving the way to systems which can monitor water use by different stakeholders near real time at various spatial scales. However, to implement an operational monitoring system based on earth observation data there needs to be established approaches with robust protocols to extract information at required spatio-temporal scale. In this study, a new approach was implemented to extract annual ET_a_ at high spatial resolution of 30 m over a large basin—LUB in Iran. The established approach demonstrates how to compute ET_a_ using a surface energy balance model over a large area covering multiple Landsat tiles of different acquisition dates and introduced a novel gap-filling approach to fill the gaps due to clouds followed by aggregate to monthly and annual maps. The monthly maps thus developed for two crop years 2013/2014 and 2014/2015 were compared with AETI data from FAO WaPOR over agriculture land use types reporting R^2^ greater than 0.9.

To logically fill the gaps in satellite derived ET_a_ maps due to clouds and stripes on Landsat 7 due to the scan line corrector failure, a combined approach of temporal interpolation followed by a spatial interpolation is recommended. The approach should also work for gapfilling other bio-physical parameters like surface temperature, vegetation indices etc., which follows a cyclic change pattern over seasons. The LWR approach to fill the gaps over time captures the temporal dynamics of ET_a_ over different agricultural land use types. This is validated by comparing with AETI data from FAO WaPOR. The approach can be extended to any other geographical area with Landsat coverage, but recommend to perform a validation analysis as demonstrated in this study before using the derived information for interventions. The open source code and documentation developed to implement this approach further facilitate the uptake by the community.

## Data Availability

The PySEBAL and the gap filling procedure developed in this study are available as an open “github” repository (url: https://github.com/wateraccounting/PySEBAL_dev) with a detailed documentation provided in “Read the Docs” technical documentation repository (url: https://pysebal.readthedocs.io/en/latest/). All datasets are disseminated as Open Geospatial Consortium (OGC) services through the project web mapping link: http://www.wa-urmia.org/.
